# Renal biopsy findings in children with FMF in Armenia: trends over the study period

**DOI:** 10.1186/1546-0096-13-S1-P88

**Published:** 2015-09-28

**Authors:** M Papazyan, H Nazaryan, A Sanamyan, N Mkrtchyan, G Amaryan

**Affiliations:** 1 “Arabkir” Medical Centre-Institute of Child and Adolescent Health, Nephrology, Yerevan, Armenia; 2 “Arabkir” Medical Centre-Institute of Child and Adolescent Health, Haemodialysis & Kidney Transplantation, Yerevan, Armenia; 3 Yerevan State Medical University, Clinical Pathology Laboratory, Yerevan, Armenia; 4 “Arabkir” Medical Centre-Institute of Child and Adolescent Health, National Paediatric Centre of Familial Mediterranean fever, Yerevan, Armenia

## 

Amyloidosis is not the only renal involvement in FMF pediatric patients.

**The purpose** of this study is to evaluate the renal biopsy results and look for the trends over the time in FMF children.

## Methods

From 1993 to 2014 renal biopsies were done in 83 FMF patients with renal involvement (mean age 12±4 year; range 2.2-18; 45 males) at “Arabkir” MC -ICAH. The diagnosis of FMF based on international clinical criteria and genetic analysis.

## Results

Renal amyloidosis (RA) revealed in 56 patients out of 83 (67%), who actually had never been treated by colchicine and FMF as well as amyloidosis were diagnosed initially on admission.

32.5% of FMF patients had other nephropathies: minimal change nephrotic syndrome (7); focal segmental glomerulosclerosis (7); acute postinfection glomerulonephritis (6); IgA nephropathy/Henoch-Schönlein nephritis (2/2); membranoproliferative glomerulonephritis type I (1); thin basement membrane disease and tubulointerstitial nephritis (1 of each).

Since 2003 the long-term program on “Early diagnosis and treatment of FMF in children in Armenia” has been implemented at National Pediatric Centre of FMF (NPC FMF) and more than 2700 children with FMF got regular colchicine therapy up to now. This resulted in *dramatic* decline of frequency of RA from 2003 to 2014: only two cases of amyloidosis have been registered in NPC FMF, while, prior to the regular colchicine treatment 16.2 % of FMF patients developed amyloidosis.

In the past 10 years a tendency of lowering the number of amyloidosis in children has been observed throughout Armenia as well (42% in 1993-2003 vs 25% in 2004-2014). Moreover during last 5 years there were only 6 pediatric cases of renal amyloidosis. Most of the patients had never received colchicine therapy.

## Conclusion

• In the last 10 years RA frequency has markedly decreased at NPC FMF due to the long-term program on improving of early diagnosis and regular colchicine treatment in FMF children. This program should be continued as the number of FMF patients is constantly increasing and amyloidosis is still a problem in children in Armenia.

• Amyloidosis is not the only type of renal involvement in FMF.

• The biopsy is mandatory in any case of renal involvement.

